# Malignant adenomyoepithelioma of the breast with axillary lymph node metastasis: a case report and review of the literature

**DOI:** 10.3389/fonc.2025.1496057

**Published:** 2025-03-06

**Authors:** Wei Wang, Long Chen, Shaoji Wen, Jianzhong Jiang, Gang Tang, Cheng Gao, Binan Wei, Feiyu Zhou

**Affiliations:** ^1^ Department of Breast and Thyroid Surgery, Ba Zhong Central Hospital, Bazhong, Sichuan, China; ^2^ Postgraduate School, North Sichuan Medical College, Nanchong, Sichuan, China

**Keywords:** malignant adenomyoepithelioma, axillary lymph node metastasis, molecular expression, case report, literature review

## Abstract

**Background:**

Adenomyoepithelioma (AME) is a relatively rare type of tumor formed by the coordinated proliferation of glandular epithelial cells and myoepithelial cells. Clinically, most of them are benign, while malignant ones are extremely rare. Malignant breast adenomyoepithelioma is somewhat invasive and may recur locally or metastasize distantly through the vascular pathway. However, reports on axillary lymph node metastasis are scarce. In this case, we present an extremely rare instance of malignant breast adenomyoepithelioma with axillary lymph node metastasis. By combining previous studies, we conduct an in-depth analysis of the clinicopathological features, treatment methods, and prognosis of this disease, and propose targeted diagnostic and treatment suggestions.

**Case presentation:**

A 64-year-old woman initially presented with no abnormalities in the axillary lymph nodes upon clinical examination or imaging. Following the diagnosis of malignant adenomyoepithelioma, the patient underwent unilateral mastectomy. Six months after surgery, the patient developed ipsilateral axillary lymph node enlargement, which was confirmed by pathological examination as metastasis of malignant adenomyoepithelioma of the breast to the axillary lymph nodes. The patient underwent axillary lymph node dissection, followed by four cycles of epirubicin and cyclophosphamide (AC) chemotherapy. Nine months after the operation, the patient returned to our outpatient clinic for follow-up, and the examination results showed no signs of recurrence or distant metastasis.

**Conclusion:**

We meticulously described the clinical symptoms, signs, and imaging characteristics of both the primary lesion of malignant adenomyoepithelioma of the breast and its axillary metastasis in the patient. Through a comparison of the pathologic features between the primary breast lesion and the axillary metastatic lymph nodes, we found a certain degree of consistency in terms of microscopic pathologic features and immunohistochemical molecular expression. Furthermore, we conducted an extensive review of the literature on breast malignant adenomyoepithelioma over the past decade. By summarizing the clinicopathological characteristics, treatment modalities, and prognosis of the patients, we found that patients with breast malignant adenomyoepithelioma have a certain risk of recurrence and metastasis. Notably, even when the clinical examination of axillary lymph nodes yields negative results and imaging shows no enlargement, a more assertive surgical approach may still be necessary. Specifically, a sentinel lymph node biopsy, despite the potential for false-negative outcomes, could be advantageous for the patient’s prognosis by enabling early determination of the need for axillary lymph node dissection.

## 1 Introduction

Adenomyoepithelioma (AME) is a tumor formed by the proliferation of both adenoepithelial and myoepithelial cells (1). Malignant adenomyoepithelioma occurs when either one or both of these cell types become malignant (2–4). When either the adenoepithelium or myoepithelium becomes malignant, it is referred to as “AME with carcinoma” in the latest version of the WHO classification of breast tumors (5). The latest WHO classification of breast tumors refers to malignant lesions of the adenoepithelium or myoepithelium as AME with carcinoma, and malignant lesions involving both adenoepithelium and myoepithelium are referred to as “epithelial-myoepithelial carcinoma” (5). Breast adenomyoepitheliomas have been reported globally as individual cases, with the majority being benign and, more rarely, malignant. Malignant adenomyoepithelioma of the breast may recur locally or metastasize remotely through blood vessels, although axillary lymph node metastasis is rarely reported. We report a 64-year-old woman who underwent total mastectomy after being diagnosed with malignant adenomyoepithelioma of the breast and developed ipsilateral axillary lymph node metastasis 6 months after surgery. We describe the clinical manifestations and imaging features of the metastatic lesion in detail, compare the pathological features of the primary breast lesion with those of the axillary metastatic lymph node, and find that there is some concordance between the two in terms of the microscopic pathological features and the expression of molecules in immunohistochemistry. We further reviewed 10 years of literature on malignant adenomyoepithelioma of the breast to summarize the clinicopathological features, treatment modalities, and prognosis of the patients. Additionally, we compared the patients in our center with three patients who had axillary lymph node metastases as reported in the previous literature, in terms of clinicopathological features, treatment modalities, and prognosis. This comparison aimed to provide reference information on the diagnosis and treatment of these rare patients, facilitating more informed clinical decision-making and potentially enhancing the management of such complex cases.

## 2 Case presentation

### 2.1 Case characteristic

**Figure 1:**
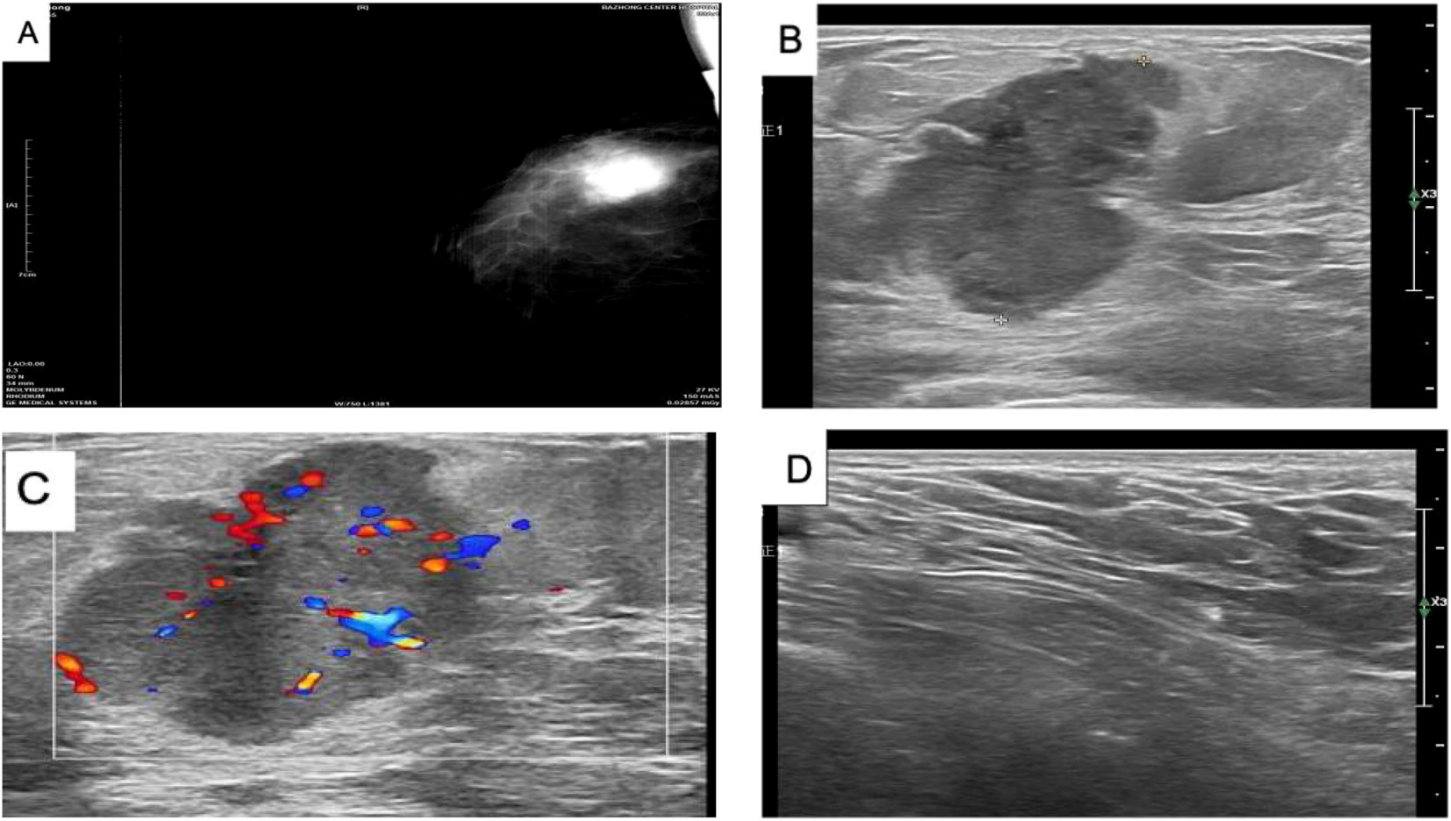
**(A)** Mammogram of the right breast showing a lobulated mass in the upper quadrant, approximately 3.8 cm × 2.5 cm in size, with irregular margins and a burr sign. No obvious calcification, BI-RADS 4B, and no enlarged lymph nodes in the axilla. **(B)** Color ultrasound showing a weakly echogenic nodule measuring approximately 28.4 mm × 27.4 mm × 31.2 mm in the upper quadrant of the right breast, with clear borders, irregular morphology, and a few irregular echoes. The nodule appeared lobulated, with a longitudinal/transversal ratio of more than 1, BI-RADS 4b. **(C)** Color ultrasound showing abundant blood flow detected within the CDEF of the right breast mass. **(D)** Color ultrasound showing no obvious enlarged lymph nodes in the right axilla.

**Figure 2:**
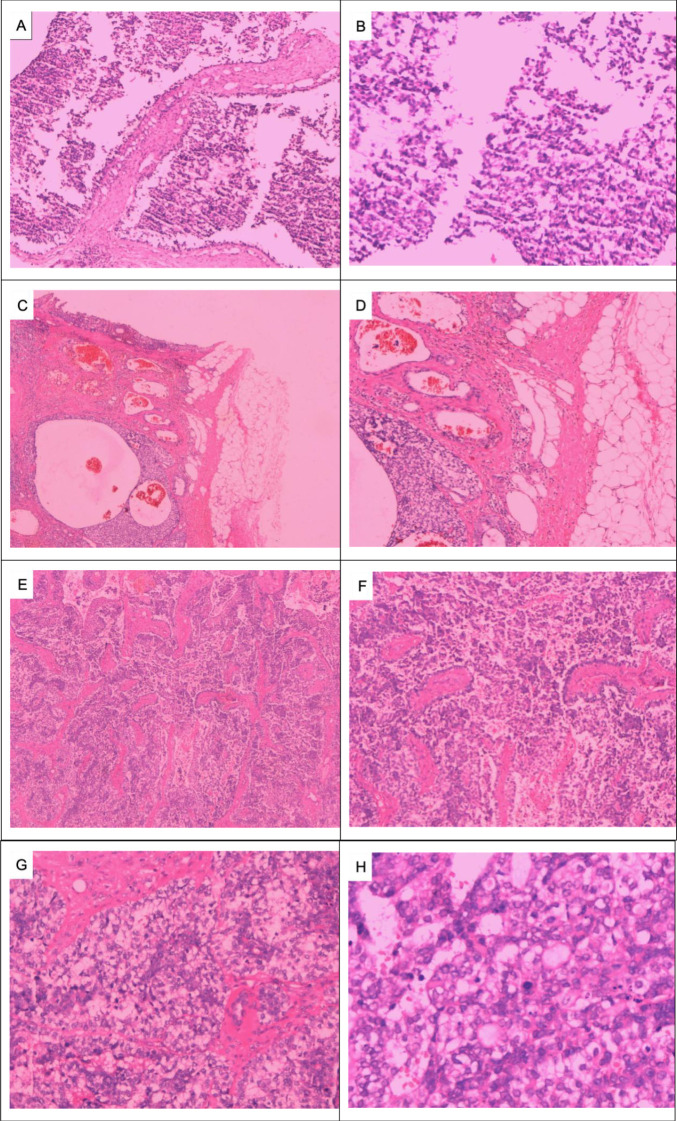
Pathologic images of the right breast mass: Puncture of the right breast mass for tissue examination showing ductal epithelial hyperplasia: **(A)** HE × 100 and **(B)** HE × 200. Right breast tumor infiltrating surrounding adipose tissue at low magnification: **(C)** HE × 40 and **(D)** HE × 100. Low-magnification view of subepithelial gland and myoepithelial hyperplasia, infiltrative tumor growth, malignant myoepithelial cells, necrosis, irregular adenoidal and solid areas, eosinophilic secretion from the gland lumen, and micropapillae: **(E)** HE × 40 and **(F)** HE × 100. Significant proliferation of translucent cells is observed at high magnification, with a mixture of deeply stained and translucent cells forming solid cell nests. The tumor cells are significantly heterogeneous, and deeply stained, with a nuclear division count of > 10/HP: **(G, H)** HE × 400).

**Figure 3:**
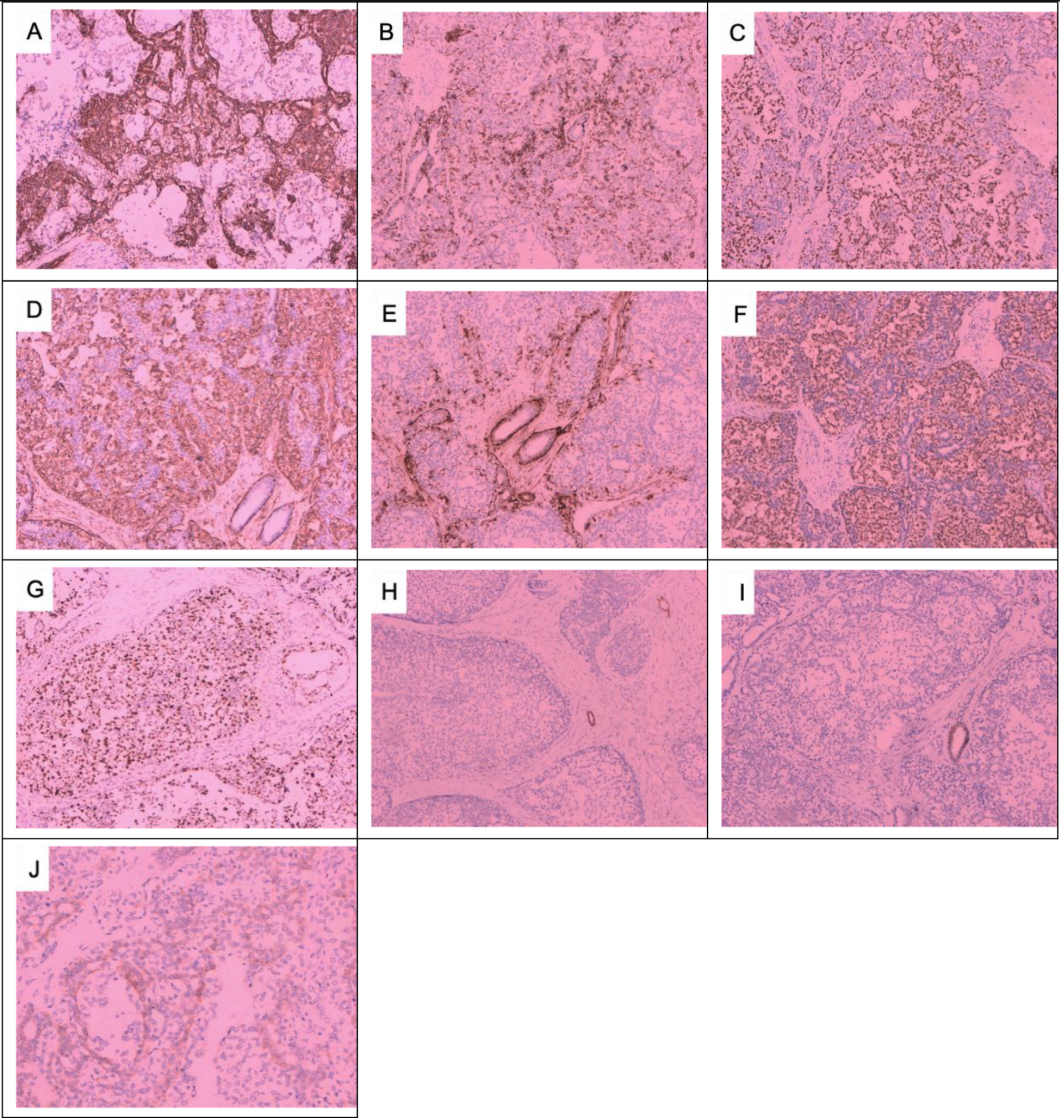
Immunohistochemical results of the right breast mass: **(A)** CK+, **(B)** CK5/6+, **(C)** P63+, **(D)** SMA+, **(E)** Caplin+, **(F)** S-100+, **(G)** Ki67 + 30%, **(H)** ER−, **(I)** PR−, **(J)** HER-2 1+. **(A–I)** × 100; **(J)** × 200.

On 29 May 2023, a 64-year-old woman was admitted to our hospital with a “1-month-old lump found in the right breast”. She did not report any additional symptoms such as breast pain or nipple discharge. The patient had her first menstrual period at the age of 14, became menopausal at age 50, had two daughters, and denied any family history of breast or ovarian cancer. Both breasts were symmetrical, with no redness or swelling of the skin, no dimpling, no nipple inversion, and a 3.5 cm × 3.0 cm × 2.5 cm mass detected in the right breast at the 10 o’clock position, 3 cm from the nipple. The mass was hard in texture, had unclear boundaries, was not easily movable, and showed no obvious adhesion to the chest wall muscles or the skin of the breasts. No obvious mass was detected in the left breast, and no enlarged lymph nodes were found in the axilla or upper and lower clavicle bilaterally. Preoperative breast ultrasound (**Figure 1**) revealed a weakly echogenic nodule measuring approximately 28.4 mm × 27.4 mm × 31.2 mm in the upper outer quadrant of the right breast. The nodule had clear borders, irregular morphology, a few irregular echoes, and a lobular shape, with a longitudinal/transversal ratio of more than 1, and was classified as BI-RADS 4b (**Figure 1B**). Abundant blood flow signals were detected within the inner part of the CDEF (**Figure 1C**), and no enlarged axillary lymph nodes were observed (**Figure 1D**). Mammography (**Figure 1A**) revealed that both mammary glands were of a small glandular type. A lobulated mass measuring approximately 3.8 cm × 2.5 cm was observed in the upper outer quadrant of the right breast, with irregular margins and a burr sign, classified as BI-RADS 4B. No enlarged axillary lymph nodes were detected. Abdominal ultrasound and bone imaging showed no abnormalities. We performed a preoperative puncture biopsy on the patient’s right breast mass, which suggested ductal epithelial hyperplasia under microscopy (**Figure 2**). On 5 June 2023, the patient underwent excision of the right breast mass at the 10 o’clock position under local anesthesia. Postoperative pathology revealed that the tumor tissue was arranged in sheets and nests under the microscope, with a visible capsule structure and double-layered cellular architecture. Most cells exhibited plasma cell translucency, visible nuclear schizonts, and lamellar necrosis. Focal areas of the margins infiltrated adipose tissue. Based on immunohistochemical findings, the diagnosis was consistent with malignant adenomyoepithelioma (see **Figure 2**). Immunohistochemistry findings (**Figure 3**): ER (−), PR (−), HER-2 (1+), E-cad (+), Pan-TPK (−), P63 partially (+), P53 wild-type, SMA partially (+), S-100 partially (+), CK (+), GATA3 little (+), CK5/6 (+), Ki-67 (+, about 30%), Vim (+), CD34 (+), calponin (+), CK (+), mammaglobin (+), and Pan-TPK (−).

**Figure 4:**
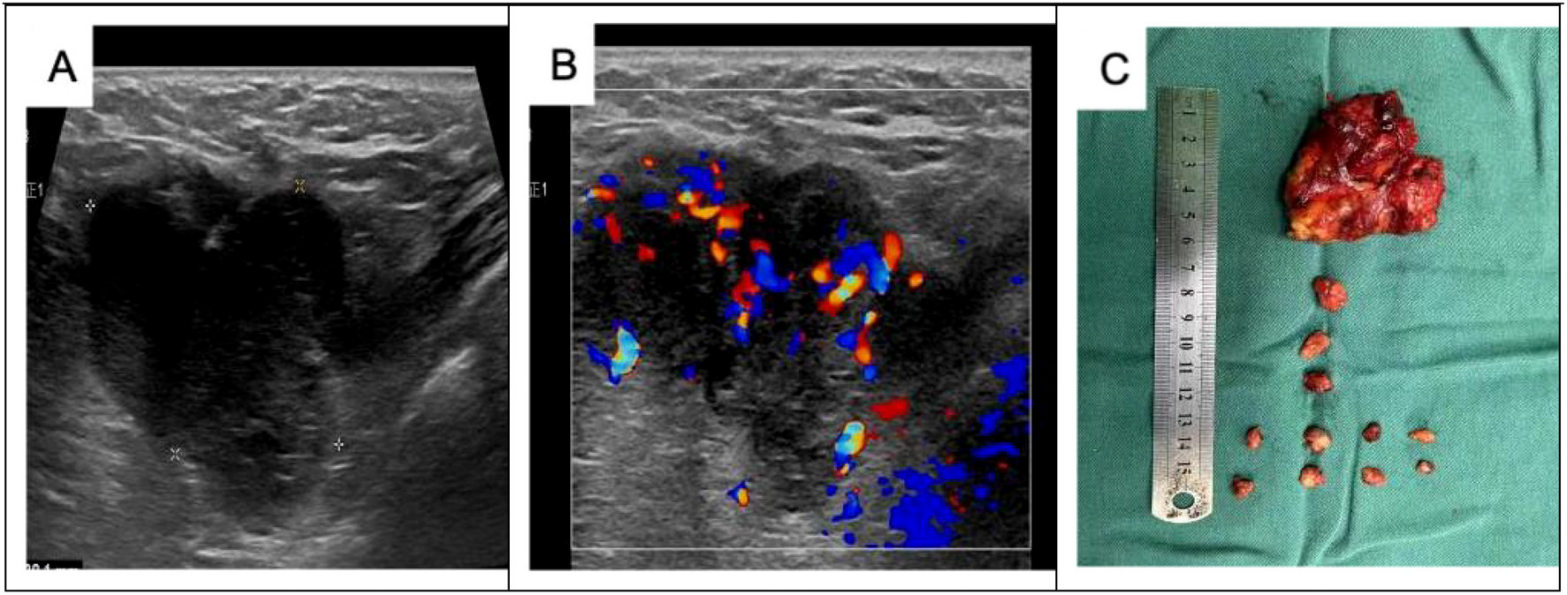
Preoperative ultrasound and postoperative images of the right axillary mass: **(A)** a 30.2 mm × 33.0 mm × 54.3 mm weakly echogenic mass detected in the right axilla, with poorly defined borders, irregular morphology, and uneven internal echogenicity. **(B)** Rich blood flow signals are detected inside the CDEF, with tortuous and irregular internal vascularization. **(C)** Thirteen axillary lymph nodes were cleared during the operation. Postoperative pathology revealed one cancer metastasis, while the remaining 12 lymph nodes showed reactive hyperplasia.

**Figure 5:**
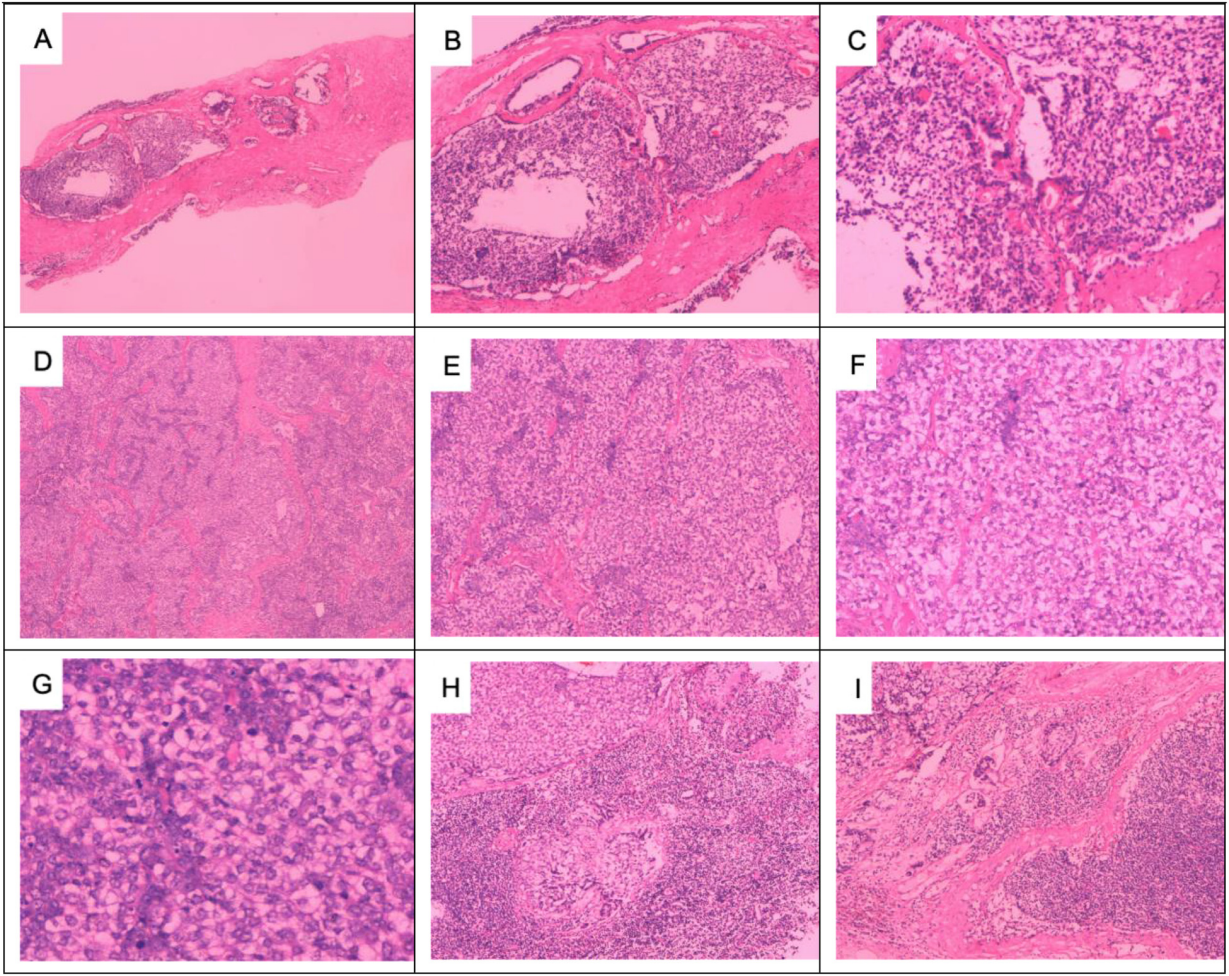
Preoperative puncture pathology and postoperative pathology of right axillary lymph node metastases: axillary lymph node puncture shows atypical cells: **(A)** HE × 40, **(B)** HE × 100, and **(C)** HE × 200. Adenomyoepithelial hyperplasia and malignant myoepithelial tumor cell infiltration in axillary lymph nodes: **(D)** HE × 40, **(E)** HE × 100, and **(F)** HE × 200. Malignant myoepithelial cells are markedly heterogeneous, with deeply stained nuclei and a nuclear division count of > 10/HP: **(G)** HE × 400. Tumor invasion destroys the margins of the lymph nodes: **(H, I)** HE × 100.

**Figure 6:**
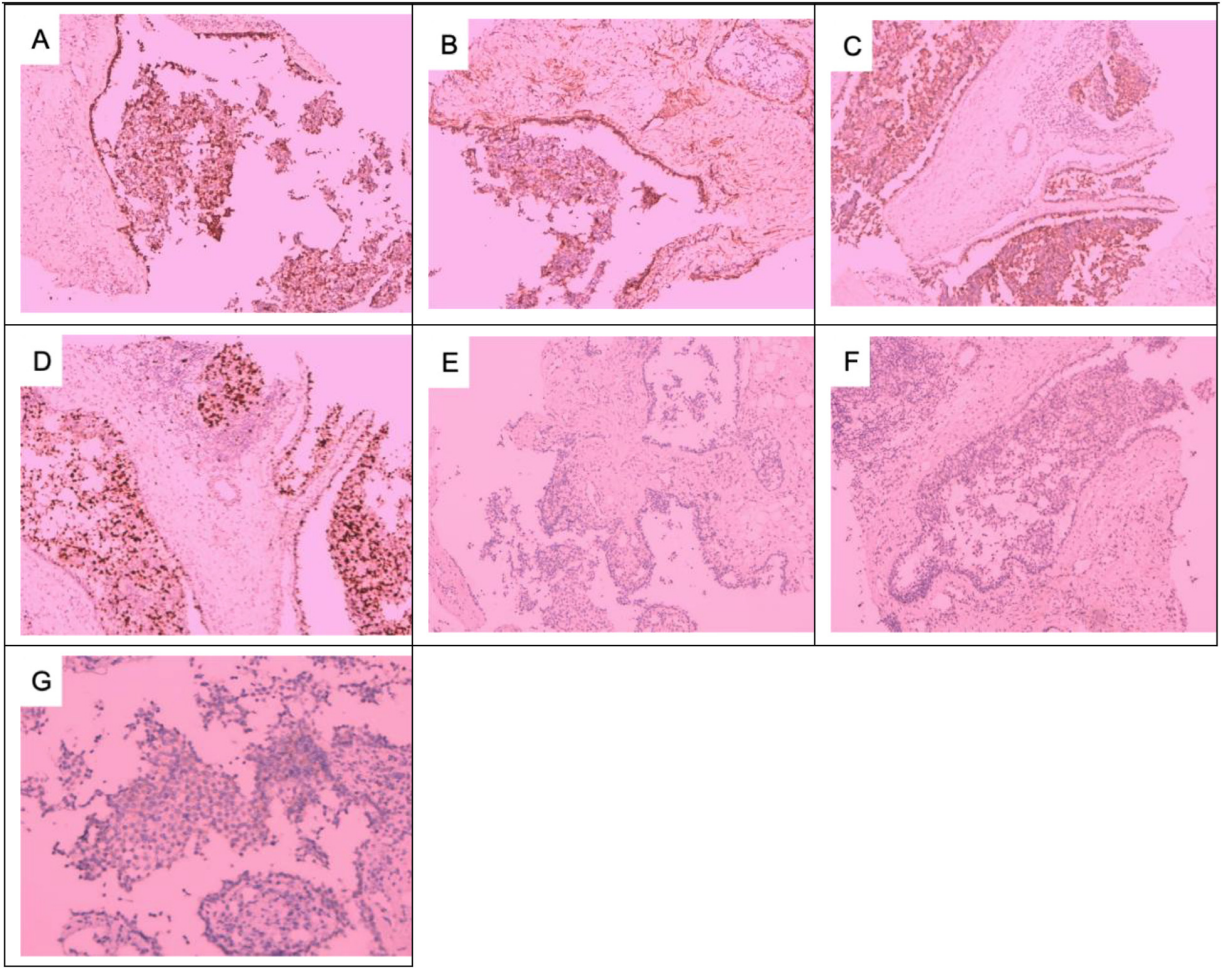
Immunohistochemical results of axillary lymph node metastases: **(A)** CK5/6+, **(B)** SMA+, **(C)** S-100+, **(D)** Ki6730%, **(E)** ER−, **(F)** PR−, and **(G)** HER-2 1+. **(A–F)** × 100; **(G)** × 200.

**Figure 7:**
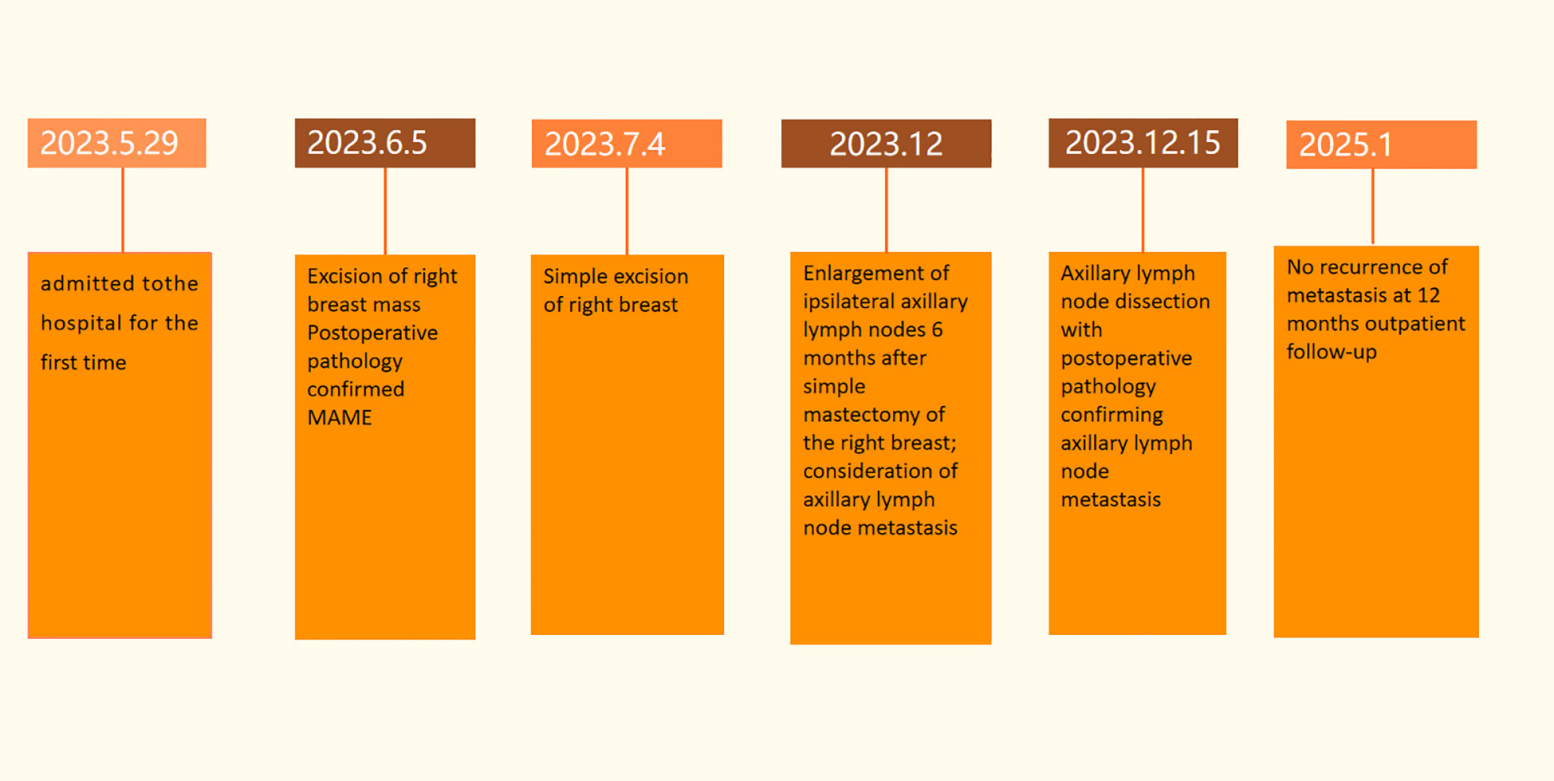
Timeline of patient consultations.

The patient was readmitted to the hospital on 3 July 2023, and following the wishes of the patient and her family, a simple right mastectomy was performed under general anesthesia on 4 July 2023, without postoperative radiotherapy. Six months after the operation, the patient was hospitalized due to a “painless mass in the right axilla”. Specialized examination revealed that the right breast was missing, the surgical incision was healed, and no mass was palpable on the right chest wall. An enlarged lymph node, approximately 3 cm × 3 cm in size, was palpable in the right axilla. It was hard, fixed, and without obvious fusion. Preoperative ultrasound (**Figure 4**) showed a 30.2 mm × 33.0 mm × 54.3 mm weakly echogenic mass in the right axilla, with poorly defined borders, irregular morphology, uneven internal echoes, abundant blood flow signals in the CDEF, and tortuous, irregular internal vascularization. Cranial and thoracic CT, abdominal ultrasound, and bone imaging revealed no abnormalities. Blood routine, biochemistry, and tumor marker assessments were also normal. Pathology of the right axillary puncture indicated atypical cells in the axillary lymph node puncture (**Figure 5**). Right axillary lymph node dissection was performed on 15 December 2023. The intraoperative lymph node map is shown in **Figure 4**, and postoperative examination along with immunohistochemistry results are presented in **Figures 5**, **6**. Diagnosis of the discharge includes the following: (1) malignant adenomyoepithelioma of the breast with metastasis to the axillary lymph nodes, (2) malignant adenomyosarcoma of the breast with metastasis to the axillary lymph nodes, and (3) malignant adenomyoepithelial tumor with metastasis to the axillary lymph nodes. Postoperative chemotherapy with epirubicin combined with a cyclophosphamide (AC) regimen was received four times. Nine months after the operation, the patient was rechecked in our outpatient clinic, and no recurrence or distant metastasis was observed. We will continue to follow-up at a later stage. The patient's entire visit timeline is shown in **Figure 7**.

This study is reported in accordance with the principles of the CARE guidelines (X).

### 2.2 Literature review

#### 2.2.1 Literature search results

We performed a literature search of PubMed using the following keyword: “malignant adenomyoepithelioma of the breast”. A total of 221 papers were retrieved and screened for studies in the last 10 years from 2014 to 2024. After screening eligible titles and abstracts, and reviewing the abstracts and headings, we ultimately included 26 studies with a total of 44 patients. For each included paper, we recorded the author(s), year of publication, demographics, clinicopathologic characteristics, treatment, and follow-up.

We further searched for all patients with malignant mammary adenomyoepithelioma (MAME) published from 1920 to 2025 and identified a total of three patients with MAME with axillary lymphatic metastasis. We summarized the clinicopathological manifestations and prognosis of the breast mass and axillary lymph nodes in these patients and compared them with the cases in our institute. This comprehensive search and comparison process allowed us to gather a wealth of data, which will prove invaluable in understanding the nuances of this rare disease and in formulating more effective diagnostic and treatment strategies.

#### 2.2.2 Characteristics of included studies

A total of 26 relevant papers involving 44 patients were published from 2014 to 2024 (**Table 1**). Subsequently, we meticulously summarized the clinical data of these patients (**Tables 2**, **3**). Notably, all the reported patients were women, with ages spanning from 34 to 78 years old. Middle-aged and elderly patients over 50 years old were predominant, accounting for 61% (39/44). Geographically, the incidence was mainly concentrated in Asian countries, making up 75% (33/44), while 16% (7/44) occurred in the Americas, 4.5% (2/44) in Europe, and 4.5% (2/44) in the Pacific region. Tumor size ranged from 0.4 cm to 7 cm. Specifically, tumors smaller than 2 cm accounted for 43.2% (19/44), those between 2 cm and 5 cm constituted 40.9% (18/44), and tumors larger than 5 cm represented 4.5% (2/44). Additionally, 11.4% (5/44) of cases had no recorded tumor size. Regarding axillary lymph node metastasis, only one out of 44 patients exhibited this condition, accounting for 2%. The expression patterns of ER, PR, and HER-2 were predominantly negative. In terms of follow-up duration, 27.3% (12/44) of patients had less than 1 year of follow-up, 50% (22/44) had follow-up between 1 year and 3 years, and 22.7% (10/44) had follow-up for more than 3 years. As for treatment modalities, MAME patients were managed with surgery, radiotherapy, and endocrine therapy. Chemotherapy was selected as an adjuvant treatment for 18.2% (8/44) of the patients, local radiotherapy for 31.8% (14/44), and endocrine therapy for 13.6% (6/44) of the patients. The overall follow-up period ranged from 0 to 132 months, with an average of 26 months. During this period, three patients experienced local recurrence, accounting for 6.8%, and eight patients had distant metastasis, accounting for 18.2%, with the lung being the most common metastatic site. Tragically, four patients died, accounting for 9.1%. This comprehensive analysis of the included studies provides crucial insights into the clinical landscape of this rare disease, facilitating more informed decision-making in both diagnosis and treatment. Regarding surgical modalities, local excision of the breast mass accounted for 31.8% (14/44). Notably, in these cases, there was no recurrence of metastasis and death. The combination of local excision and sentinel lymph node biopsy was adopted in 13.6% (6/44) of the patients, among whom one patient unfortunately experienced local recurrence and lung metastasis. Breast-conserving surgery combined with sentinel lymph node biopsy accounted for 9.1% (4/44), and breast-conserving surgery alone also accounted for 9.1% (4/44). Interestingly, in the reports, the one patient who underwent either of these two surgical procedures experienced no local recurrence, distant metastasis, or death. Total breast excision accounted for 9.1% (4/44). In this group, one patient had local recurrence and lung metastasis, but no deaths were reported. The combination of total breast excision and sentinel lymph node biopsy was performed in 29.6% (13/44) of the cases. in this group, one patient died due to cardiac, pulmonary, renal, and cerebral metastasis, another patient succumbed to lung metastasis, while a third patient, who had multiple liver, pleural, and abdominal wall metastases, was still alive at the time of the report. Additionally, one patient with lung metastasis was also alive at that time, and there were no recurrence cases in this subset. Modified radical surgery was carried out in 4.6% (2/44) of the cases. Unfortunately, one patient in this category died after developing local recurrence and pulmonary metastases. Radical surgery was performed in 2.2% (1/44) of the cases, and the patient died during follow-up. For axillary lymph node management, a significant number, 23 patients (52%), underwent sentinel lymph node biopsy. Remarkably, none of these patients had sentinel lymph node metastasis, and no axillary lymph node metastasis was detected during subsequent follow-up. Meanwhile, three patients underwent axillary lymph node dissection, all of whom had previously undergone modified radical surgery. In one particular case, the patient was diagnosed with MAME after mass resection, and axillary lymph node dissection was performed in the second month of the postoperative period once axillary lymph node metastasis was identified. This detailed breakdown of surgical and axillary lymph node management strategies offers a comprehensive overview of current clinical practices in treating this rare disease, assisting in the evaluation and potential optimization of future treatment approaches.

**Table 1:** Summary of case reports on malignant adenomyoepithelioma of the breast: clinical information and pathological characteristics over the past 10 years.

Literature	Cases/region	Age (the average)	Tumor size (cm)	Lymph node metastasis	ICH (ER/PR/HER2)	Treatment	Follow-up/survival time (month)	Recurrence/site of metastasis
Nasrollah Ahmadi et al. (2015) (6)	1	46	1.9	No	−/−/NA	Mastectomy	24	No
Kim et al. (2019) (7)	1	56	1.5	No	−/−/−	Wide excision	24	No
Kazuki Moro et al. (2020) (3)	1	64	3.4	No	NA	Mastectomy and SLNB + chemotherapy	17	Brain, heart, lung1, kidney
Goshi Oda et al. (2021) (8)	1	53	2	No	NA	Mastectomy + partial resection of the pectoralis muscle	36	No
Longqing Hu et al. (2022) (9)	1	55	3	No	−/−/−	Modified radical mastectomy + chemotherapy	24	No
Heba Mohammad et al. (2022) (10)	15	34–67/50	0.4–7/1.8	No	(NA/NA/NA) 4 (−/−/−) 5 (+/+/−) 3 (−/−/+) 1 (+/−/−) 1 (+/+/NA) 1	Lump excision: 9 Lump excision + SLNB: 2 Mastectomy + SLNB: 2 Breast-conserving surgery + SLNB: 2 Radiotherapy: 10 Chemotherapy: 3 Endocrine therapy: 5	5–162	No 1r + lung2 1 lung3 die
Zhihao Zhang et al. (2021) (11)	1	64	4.5	No	−/−/−	Mastectomy and SLNB	12	No
Antonelli et al. (2018) (12)	1	72	3	No	−/−/−	Upper left quadrantectomy surgery + SLNB	12	No
Puja Parikh et al. (2021) (13)	1	61	1.5	No	−/−/−	Lump excision + SLNB4	0	No
Kishore Reddy Bojja et al. (2022) (14)	1	70	5	No	NA	Wide excision of the lump	0	No
Duan-Yang Zhai et al. (2021) (15)	2	46	2	No	−/−/−	Mastectomy + SLNB + chemotherapy	24	No
		58	4	No	−\−\−		4	
Naomi Wiens et al. (2019) (16)	3	58 (43–66)	NA	No	NA	Lump excision + SLNB: 2 Mastectomy: 1	61 7 15	1 No 1 No 1 died of metastatic disease
Zhu Yuan et al. (2017) (17)	2	58	3	Yes	NA	Radical mastectomy + radiotherapy + chemotherapy:	35	1 died fpleural effusion
		51	3	No	−\−\−	Mastectomy + SLNB: 1	21	1 alive
August W. Moritz et al. (2016) (18)	1	78	1.7	No	−/−/NA	Mastectomy + SLNB	12	Alive
Agnieszka Korolczuk et al. (2016) (19)	1	47	2.6	No	−/−/NA	Mastectomy + SLNB	31	Alive Lung5
Jing Xu et al. (2016) (20)	2	54	2.3	No	−/−/NA	Lumpectomy	60	No alive
		48	2	No	+/+/−	Breast-conserving surgery + SLNB	96	No alive
Suee Lee et al. (2015) (21)	1	51	NA	No	−/−/−	Mastectomy with axillary lymph node dissection + chemotherapy	10	Multiple hepatic, pleural, and abdominal wall
Ying Yang et al. (2014) (22)	1	61	1.5	No	−/−/−	Modified radical mastectomy	12	Alive
Jones M et al. (2017) (23)	1	78	1.7	No	−/−/NA	Lumpectomy → mastectomy + SLNB	12	Alive
Natalie Logie et al. (2017) (24)	1	63	2.4	Yes	NA	Mastectomy with lymph node dissection and adjuvant radiotherapy	3	Alive
Lucy Elizabeth Hempenstall MBBS, MPH1 (2018) (25)	1	45	6.5	No	−/−/−	Lumpectomy = radiotherapy + chemotherapy	0	Alive
Satomi Watanabe (2019) (26)	1	41	UK	No	−/−/−	Mastectomy radiotherapy	0	Lung
Kakkar et al. (2019) (27)	1	36	1.8	No	+/+/−	Lumpectomy + SLNB	12	No
Febres-Aldana et al. (2020) (28)	1	47	2.8	No	NA/NA/NA	Lumpectomy	12	No
Lari et al. (2020) (29)	1	39	3	No	−/−/−	Lumpectomy → mastectomy + SLNB	0	No

**Table 2:** Summary of data from 44 patients.

Characteristics	All patients n=44
Age (year)	
≦50	17 (38.6)
> 50	27 (61.4)
Region	
Asia	33 (75)
America	7 (16)
Europe	2 (4.5)
Oceania	2 (4.5)
Size	
< 2 cm	19 (43.2)
2–5 cm	18 (40.9)
> 5 cm	2 (4.5)
Not recorded	5 (11.4)
Axillary lymph node metastasis	
Yes	1 (2.2)
No	43 (97.8)
ER	
+	8 (18.2)
−	26 (59.1)
Not recorded	10 (22.7)
PR	
+	6 (13.6)
−	28 (63.7)
Not recorded	10 (22.7)
HER-2	
+	1 (2.3)
−	26 (59.1)
Not recorded	17 (38.6)
Follow-up time (year)	
< 1	12 (27.3%)
1–3	22 (50.0)
> 3	10 (22.7)

**Table 3:** Treatment and prognosis.

Characteristics	All patients n=44
Surgery	
Lumpectomy	14 (31.8)
Lumpectomy + SLNB	6 (13.6)
Breast-conserving surgery + SLNB	4 (9.1)
Mastectomy	4 (9.1)
Mastectomy and SLNB	13 (29.6)
Modified radical mastectomy	2 (4.6)
Radical mastectomy	1 (2.2)
Radiation therapy	
Yes	14 (31.8)
No	30 (68.2)
Chemotherapy	
Yes	8 (18.2)
No	36 (81.8)
Hormonal therapy	
Yes	6 (13.6)
No	38 (86.4)
Recurrence	
Yes	3 (6.8)
No	41 (93.2)
Survival status	
Died	4 (9.1)
Alive	40 (90.9)
Metastasis	
Yes	8 (18.2)
No	36 (81.8)

We collected clinical data on four patients who presented with axillary lymph node metastasis (**Table 4**). In 1994, Chen et al. reported a case in which a patient with MAME was found to have a right breast mass and enlarged axillary lymph nodes during the initial consultation. Preoperative puncture indicated the presence of atypical cells in both the breast mass and axillary lymph nodes. Subsequently, the patient underwent mastectomy and axillary lymph node dissection but did not receive relevant adjuvant treatment. Unfortunately, 7 months later, the patient developed bone metastasis. In 2007, Suresh Attili et al. reported a case of MAME. At the time of initial diagnosis, a right breast mass was detected, though the axillary status was not elaborated upon. A mastectomy was performed, but 2 months after the operation, the patient experienced local recurrence and enlargement of the axillary lymph nodes on the same side. Another mastectomy was then performed along with clearance of the axillary lymph nodes. After the operation, a definitive diagnosis of MAME with metastasis to the axillary lymph nodes was confirmed, and postoperative radiotherapy was administered. During the 18-month follow-up, the patient had lung metastasis. Zhu Yuan et al. reported a patient who was admitted for a lumpectomy of a left breast mass. Postoperative pathology confirmed MAME. During the follow-up recall, the patient underwent radical surgery but exhibited poor compliance. Eleven months later, the patient’s left breast ulcerated, and axillary lymph node enlargement was observed. Radical surgery was performed again. Sadly, 2 years after the operation, the patient died from bone metastasis. Pathologically, among the three patients with combined axillary lymph node metastasis, the malignant component of the primary breast lesion was consistent with that of the axillary lymph node metastasis site, while in one case, this was not described. In one patient, the expression of ER and PR was inconsistent between the breast mass and axillary metastasis. This compilation of detailed patient cases further enriches our understanding of the complex clinical manifestations and pathological features associated with MAME and axillary lymph node metastasis, providing valuable insights for future diagnosis and treatment strategies.

**Table 4:** Clinicopathological characteristics and prognosis of four patients with MAME complicated by axillary lymph node metastasis.

Case	Age (year)	Tumor size (cm)	Physical examination	Imaging	Puncture cytology			
1. Chen (1994) (30)	47	1.7	Breast mass + enlarged lymph nodes	Breast mass + enlarged lymph nodes	Atypical cells			
2. Suresh Attili et al. (2007) (31)	20	NA	Breast mass	Breast mass	Malignant cells			
3. Zhu Yuan (2017) (17)	58	3	Breast mass	NA	Malignant cells			
4. Our study (2025)	63	3.5	Breast mass	Breast mass	Atypical cells			
Case	Surgery	Adjuvant therapy	Follow-up/survival time (month)	Recurrence	Metastasis	Outcome		
1	M + L	No	7	No	Bone, axillary lymph nodes	Died		
2	EM → MRM	Radiochemotherapy	18	Yes	Axillary lymph nodes, lung	Alive		
3	EM → MRM	Chemotherapy	24	Yes	Axillary lymph nodes, bone	Died		
4	M → RM	Chemotherapy	12	No	Axillary lymph nodes	Alive		
Case	MCBM	ER^a^	PR^a^	HER-2^a^	MCLN	ER^b^	PR^b^	HER-2^b^
1	M	NA	NA	NA	M	NA	NA	NA
2	E + M	−	−	−	E + M	NA	NA	NA
3	NA	−	+	−	NA	+	−	−
4	M	−	−	−	M	−	−	−

*M*, mastectomy; *ML*, mastectomy + lymph node dissection; *EM*, excision of the mass; *MRM*, modified radical mastectomy; *E*, malignant transformation of epithelium; *RM*, radical mastectomy; *M*, malignant transformation of myoepithelium; *MCBM*, malignant components of breast masses; *MCLN*, malignant components of lymph node.

a Breast tumor: ER, PR, and HER-2.

b Lymph node: ER, PR, and HER-2.

## 3 Discussion

MAME is an extremely rare breast tumor, with most relevant literature consisting of case reports and reviews. We reviewed the reported cases of MAME from the past 10 years (**Table 1**). Notably, all the patients were women, with an age range of 34 to 78 years. The majority of cases were in middle-aged and older individuals, specifically those over 50 years old, who accounted for 61% of the cases (**Table 1**), corroborating the findings of previous studies (17, 31, 32). Interestingly, our analysis revealed that the incidence of MAME was predominantly in Asia (33/44), constituting 75% of the cases. This preponderance in Asia may be attributed to the large Asian population. Given that countries like China and India are highly populous, it is plausible that rare diseases are reported more frequently in these regions. However, whether this distribution is related to ethnicity remains uncertain, as there are currently no relevant reports. Thus, further genetic research is needed to elucidate this aspect. This new understanding of the demographic and geographical patterns of MAME incidence provides a foundation for future research directions and potential public health interventions.

MAME predominantly occurs in women, although isolated cases of adenomyoepithelioma of the breast in men have been reported (33). Similar to breast cancer, the site of malignant breast AME is typically in the upper outer quadrant of the breast (9, 34). In the present case, the tumor occurred in the right breast at the 10 o’clock position, 3 cm from the nipple, which is consistent with previous reports.

In the classification of breast tumors published by the World Health Organization in 2019, AME is defined as epithelial-myoepithelial lesions of the breast (5), which are microscopically characterized by the proliferation of epithelial and myoepithelial cells in the ducts and/or tubules of the breast (24, 32, 36–38). Tavassoli describes malignant AME tumors as having a storiform, tubular, and lobular histological pattern (35), and the present case is consistent with the storiform histologic type, formed mainly by myoepithelial cells with a small amount of glandular epithelial proliferation. The diagnosis of malignant AME generally requires the inclusion of the following features: increased nuclear schizophrenia (> 3/10 HPF), cellular anisotropy, necrosis or metastasis of the tumor, and an infiltrative growth pattern (24, 36–38). In our case, the right breast mass exhibited microscopic hyperplasia of myoepithelial cells and glandular epithelium within the tumor tissues. Most of the myoepithelial cells showed translucent cytoplasm, with nuclear schizophrenia and sheet-like necrosis. The nuclear division count was greater than 10/HPF, and focal areas at the margins infiltrated adipose tissue. The malignant component was identified as myoepithelial cells, which was consistent with the diagnosis of MAME. Immunohistochemistry markers such as CK and CK5/6 are used for adenoepithelial identification, while SMA, S-100, P63, calponin are myoepithelial markers. The positivity of both marker types supports the diagnosis of AME (7). In this case, immunohistochemistry showed positive expression of CK, CK5/6, P63, SMA, calponin, and S-100, which further supported the diagnosis of AME.

Our patient was negative for HER-2 (1+), ER, and PR. Notably, most reports in the literature indicate that the majority of MAME cases show negative expression for these molecular markers (18, 39), which is consistent with the findings in the 44 patients we summarized. As a result, our patient did not have the option for targeted and endocrine therapy. Upon reviewing the treatments for the 44 patients, we found that radiotherapy and chemotherapy were not commonly used treatment modalities. This may be attributed to patient compliance and physicians’ awareness of MAME. It is important to emphasize that MAME, as a malignant tumor, harbors a certain risk of recurrence and metastasis. Some patients, particularly those who did not undergo timely lumpectomy, experienced recurrence and metastasis, and some even succumbed within a short period. Currently, there is a lack of prospective clinical studies on malignant adenomyoepithelioma of the breast. The rarity of cases makes it challenging to establish clear systemic treatment modalities and protocols, with only a few retrospective case studies available. For instance, Waqar Haque et al. reviewed the data of 110 MAME cases (40), with a median follow-up of 52 months and an OS rate of 74.4%. Multifactorial analysis indicated that surgical modality, chemotherapy, radiotherapy, and endocrine therapy were not correlated with overall survival in patients. Moreover, the effect of radiotherapy in patients with recurrence, metastasis, or fatal outcomes remains unclear. Therefore, treatment plans for MAME patients generally follow those for breast cancer. Our recommendation is not to forgo chemotherapy, targeted therapy, or endocrine adjuvant therapies for MAME patients. Additionally, local radiotherapy can be considered for patients with axillary lymph node metastasis following axillary lymph node dissection. This comprehensive analysis and proposed treatment strategy aim to optimize the management of MAME patients, considering the current state of knowledge and clinical challenges.

MAME typically manifests as painless breast lumps, with pain and nipple discharge being rare symptoms (41, 42). Local recurrence and distant metastasis can occur, with metastatic sites predominantly involving the lungs, brain, and bones. Notably, hematogenous metastasis is more commonly documented than lymph node metastasis. Through our summary, we found that the lungs are the most common metastatic site among patients, similar to breast cancer. Currently, axillary lymph node metastasis in MAME is widely regarded as extremely rare (7). Upon reviewing the literature, we identified only three reported cases of axillary lymph node metastasis (17, 31, 32). Regrettably, the reported cases did not provide detailed information on the clinical symptoms and signs, imaging features, pathologic features, and molecular expression of the axillary lymph node metastatic lesions. This lack of detail hinders a comprehensive understanding and further research into this particular aspect of MAME, underscoring the need for more in-depth investigations in future studies. Zhu Yuan et al. reported cases showing inconsistency in ER and PR expression between primary breast tumors and axillary lymph node metastases (17), suggesting the need for physicians to re-evaluate the pathology and molecular expression of recurrent metastases in patients with MAME. This approach can help determine whether the patient requires additional targeted, endocrine, or chemotherapeutic treatments, similar to those used for recurrent metastatic breast cancer. We reviewed 44 patients with MAME and found a lack of uniformity in breast surgery approaches. Alarmingly, local recurrence has been reported even in patients who underwent total mastectomy, while those who had only lumpectomy experienced local recurrence or even axillary lymph node metastasis. Therefore, we recommend that patients with a pathologically confirmed diagnosis of MAME should preferably undergo total mastectomy. However, if the patient strongly desires breast conservation, the indications for breast-conserving surgery should be carefully evaluated to determine whether breast conservation is appropriate, while always ensuring safe margins. For patients with MAME, there are currently no relevant guidelines or consensus regarding axillary lymph node surgery in those without axillary lymph node metastasis as indicated by preoperative examination and imaging. MRI can be beneficial for detecting tiny axillary lymph node foci, but it should take the patient’s economic situation into account, as it may not be feasible for every patient. We summarized the management of axillary lymph nodes in patients with MAME from the literature. Among 44 patients, 23 underwent sentinel lymph node biopsy, and remarkably, none of the patients had sentinel lymph node metastasis. Furthermore, no axillary lymph node metastasis was detected in those who were later followed up. Three patients underwent axillary lymph node dissection, one of whom had the procedure after axillary lymph node metastasis was detected 2 months following a clear localized resection of the mass. Therefore, we recommend that patients with enlarged lymph nodes on preoperative check-ups or imaging undergo routine axillary lymph node biopsy before surgery. For those in whom malignant cells are clearly identified, axillary lymph node dissection should be performed. In conclusion, we advocate for axillary lymph node dissection in patients with atypical cells or benign lesions, based on preoperative routine sentinel lymph node biopsy and enlarged lymph node excision biopsy, as well as in those with confirmed cancer metastasis. This comprehensive set of recommendations aims to optimize surgical management and overall treatment outcomes for patients with MAME, considering the current clinical challenges and uncertainties.

In this case, preoperative examinations, including ultrasound and molybdenum target imaging, suggested no metastasis in the axillary lymph nodes. The patient was diagnosed with MAME and underwent a right mastectomy without axillary sentinel lymph node biopsy and dissection. However, the patient developed significant axillary lymph node enlargement within the first 6 months after the operation. A puncture biopsy confirmed MAME, leading to subsequent axillary lymph node dissection. Ahlam et al. (43) reported a case of malignant AME in which preoperative examination did not reveal enlarged axillary lymph nodes, although the results of axillary lymph node imaging were not described. Intraoperative biopsy of the sentinel lymph nodes revealed axillary lymph node metastasis. This case suggests that after the diagnosis of MAME, surgical approaches to axillary lymph nodes may be relatively conservative. For patients with no axillary lymph node enlargement indicated by preoperative examination and imaging, however, a more aggressive surgical approach to axillary lymph node management may be warranted. Currently, breast conservation with sentinel lymph node biopsy or simple mastectomy with sentinel lymph node biopsy is commonly utilized for early-stage breast cancer. The driving force behind this preference is that axillary lymph node dissection invariably ushers in a multitude of postoperative complications, such as edema, pain, and fluid retention. When managing MAME patients, it is equally important to consider the potential complications that axillary lymph node dissection may cause. Moreover, it remains equivocal whether axillary lymph node dissection confers any benefits in terms of curtailing local recurrence and enhancing overall survival for these patients. Given this uncertainty, we firmly do not recommend routine axillary lymph node dissection for patients who present no signs of axillary lymph node metastasis during preoperative examination and imaging. This stance is adopted to shield patients from superfluous risks and to fine-tune the treatment regimen, considering both the potential downsides of the procedure and the lack of conclusive evidence supporting its routine application in such scenarios. It is in harmony with the overarching principle of optimizing patient care by minimizing harm and maximizing therapeutic gains.

The current literature reports that most benign AMEs have a favorable prognosis. However, in the case of MAME, local recurrence and distant metastasis can occur, and the prognosis of recurrent metastatic MAME is extremely poor, with most deaths happening within a few years after the initial treatment (10, 17). The lack of sufficient follow-up time and subsequent reports indeed hampers the accurate presentation of the results. We summarized the prognosis of 44 patients with MAME reported over the past 10 years. Among them, three patients had local recurrence, eight patients suffered from distant metastasis, and four patients died. It should be noted, though, that some of the reports had a relatively short follow-up period, leaving the subsequent survival of patients unknown. This inevitably affects our comprehensive understanding of the disease’s prognosis. In a review of the three patients who developed axillary lymph node metastasis in the past, two of them went on to develop bone metastasis. One case died after 7 months of survival, and another died after 24 months. These findings seem to strongly suggest that patients with MAME presenting with axillary lymph node metastasis generally have a rather bleak prognosis. In the current case under discussion, the breast mass diameter of the MAME patient was greater than 3 cm and axillary lymph node metastasis was present, indicating a certain risk of local recurrence and distant metastasis. Therefore, we will provide health education to the patient to encourage active treatment and ensure regular follow-up. This proactive approach is crucial in managing the patient’s condition and potentially improving their long-term outcomes, given the challenges and uncertainties associated with MAME.

## 4 Conclusion

This study comprehensively explores breast malignant adenomyoepithelioma with axillary lymph node metastasis. Firstly, we provide a detailed account of the clinical symptoms, signs, and imaging features of both the primary breast lesion and axillary metastasis. Through comparison, a concordance was identified between the microscopic pathological features and immunohistochemical molecular expression of the primary breast lesion and the axillary metastatic lymph node. Subsequently, an extensive review of the literature on breast malignant adenomyoepithelioma was conducted. We summarized the clinicopathological features, treatment modalities, and prognosis of relevant patients, as well as the clinicopathological manifestations and prognosis of breast masses and axillary lymph nodes in patients with axillary lymph node metastasis, making comparisons with our reported cases.

Finally, key suggestions for treating these patients were proposed:

1. Diagnosis: for MAME patients, preoperative physicians should conduct detailed breast examinations by specialists, including routine breast ultrasound and mammography of the primary breast focus, axillary region, and systemic organs. Given the diagnostic challenges, puncture biopsy usually requires histopathology combined with immunohistochemistry for confirmation.
2. Surgical treatment: total mastectomy is an option for MAME patients. If breast conservation is desired, ensuring the safety of the breast mass and axillary lymph nodes is crucial. For patients without axillary lymph node enlargement preoperatively, a more assertive surgical approach to the axillary lymph nodes is recommended, such as sentinel lymph node biopsy. For patients with preoperative lymph node enlargement, routine axillary lymph node aspiration biopsy should be performed before surgery. Axillary lymph node clearance is necessary when malignant cells are clearly identified. For atypical or benign cells, detection and confirmation through histology combined with immunohistochemistry are required. For patients with atypical cells or benign lesions, intraoperative sentinel lymph node biopsy and excisional biopsy of enlarged lymph nodes are advised. Axillary lymph node dissection is indicated for metastatic cancer.
3. Adjuvant therapy: for patients with recurrence, metastasis, or fatal outcomes, the therapeutic effect of radiotherapy remains unclear. Based on patients’ wishes and informed consent, those with indications may opt for chemotherapy, targeted therapy, and endocrine adjuvant therapies. Timely assessment of each patient’s treatment response is essential. Additionally, local radiotherapy may be considered for patients with axillary lymph node metastasis following axillary lymph node dissection.
4. Follow-up: in MAME patients, especially those with local recurrence, axillary lymph node metastasis, or distant metastasis, both physicians and patients should heighten awareness of the disease. Encouraging patient follow-up is crucial to enhance adherence to the diagnosis and treatment process, ultimately improving patient outcomes.

## Data Availability

The original contributions presented in the study are included in the article/supplementary material. Further inquiries can be directed to the corresponding author.
